# Supplementary material for: IVIM-DWI-Based Radiomics for Lesion Phenotyping and Clinical Status Prediction in Relapsing–Remitting Multiple Sclerosis
Source: J Clin Med. 2025 Sep 24;14(19):6753. doi: 10.3390/jcm14196753 (PMC12524992; doi:10.3390/jcm14196753)
Supplement: Supplementary file 1 [file jcm-14-06753-s001.zip › jcm-3878015-supplementary.pdf]

Table S1: Supplementary Table S1. Radiomics feature-extraction parameters per IVIM map (*f*, *D*, *D**).

Category	Parameter	IVIM-*f*	IVIM-*D*	IVIM-*D**	Notes
Software & Standards	Platform/toolkit	3D Slicer v5.6.1 + PyRadiomics (IBSI-compliant)			Same toolchain used for all maps
Software & Standards	IBSI compliance	Yes	Yes	Yes	Feature definitions follow IBSI
ROI & Registration	Lesion ROIs	Manual on post-contrast T1w; mapped to IVIM maps			Single neuroradiologist; consensus review
ROI & Registration	Registration	Rigid/affine to IVIM space			Boundary-based/standard tools
Preprocessing	Bias-field correction	N4ITK: Yes	N4ITK: Yes	N4ITK: Yes	
Preprocessing	Resample voxel size	1.0 × 1.0 × 1.0 mm^3	1.0 × 1.0 × 1.0 mm^3	1.0 × 1.0 × 1.0 mm^3	
Preprocessing	Interpolation (image/labels)	B-spline/nearest neighbour	B-spline/nearest neighbour	B-spline/nearest neighbour	
Preprocessing	Intensity normalisation	Per-image normalisation	Per-image normalisation	Per-image normalisation	Applied prior to discretization
Discretization	Type	Fixed bin width	Fixed bin width	Fixed bin width	
Discretization	Bin width	0.01 (unitless fraction)	0.05×10^{-3} mm^2/s	0.5×10^{-3} mm^2/s	Aimed at ~64 grey levels per ROI
Texture (3D)	GLCM settings	13 dirs; distances {1,2}; symmetric, normalised			Directional averaging (3D)
Texture (3D)	GLRLM/GLSZM/ GLDM/NGTDM	PyRadiomics 3D defaults	PyRadiomics 3D defaults	PyRadiomics 3D defaults	Parameters kept at defaults unless specified
Filters	Wavelet	Coiflet-1; 8 sub-bands (LLL..HHH)	Coiflet-1; 8 sub-bands	Coiflet-1; 8 sub-bands	
Filters	Gaussian/LoG	σ = 1.0, 2.0, 3.0 voxels	σ = 1.0, 2.0, 3.0 voxels	σ = 1.0, 2.0, 3.0 voxels	
Feature Families	Computed	First-order, shape (3D), texture	First-order, shape (3D), texture	First-order, shape (3D), texture	Computed on original + filtered images
Robustness & Selection	Reproducibility (ICC)	ICC > 0.80 retained	ICC > 0.80 retained	ICC > 0.80 retained	Inter-observer subset (n=30 lesions)
Robustness & Selection	Dimensionality reduction	Mutual information; optional PCA (tuned)	Mutual information; optional PCA (tuned)	Mutual information; optional PCA (tuned)	Within CV; no leakage

Note: IVIM = intravoxel incoherent motion; *f* = perfusion fraction; *D* = diffusion coefficient; *D** = pseudo-diffusion coefficient; ROI = region of interest; N4ITK = N4 bias field correction (ITK); GLCM = grey-level co-occurrence matrix; GLRLM = grey-level run-length matrix; GLSZM = grey-level size-zone matrix; GLDM = grey-level dependence matrix; NGTDM = neighbourhood grey-tone difference matrix; LoG = Laplacian of Gaussian; ICC = inter-class correlation coefficient
